# Dependence in Activities of Daily Living as a Predictor of In-Hospital Mortality During COVID-19 in Older Individuals

**DOI:** 10.3390/life15020271

**Published:** 2025-02-11

**Authors:** Jan Ilkowski, Katarzyna Wieczorowska-Tobis, Przemyslaw Guzik

**Affiliations:** 1 Emergency Medicine, Poznan University of Medical Sciences, 60-608 Poznan, Poland; 2 Geriatric Unit, Department of Palliative Medicine, Poznan University of Medical Sciences, 61-245 Poznan, Poland; kwt@tobis.pl; 3 Department of Human Nutrition and Dietetics, Poznań University of Life Sciences, 60-624 Poznan, Poland; 4 Department of Cardiology-Intensive Therapy, Poznan University of Medical Sciences, 60-355 Poznan, Poland; pguzik@ptkardio.pl; 5 University Centre for Sports and Medical Studies, Poznan University of Medical Sciences, 60-802 Poznan, Poland

**Keywords:** activities of daily living, older individuals, in-hospital mortality, COVID-19

## Abstract

Activities of Daily Living (ADL) are fundamental tasks for individuals to manage their basic needs. Our study aims to examine ADL at admission (adADL) and the Pre-COVID-19 to Admission ADL Difference (ADL-change) as potential predictors of in-hospital mortality. This is a retrospective analysis of clinical data (including the Katz index for ADL) from 141 older patients aged at least 65 years hospitalized in a COVID-19-dedicated unit (not requiring ICU) from September 2021 until January 2022 in Poznań, Poland. Thirty patients (21.3% of all) died during hospitalization. Non-survivors were older than survivors, exhibited lower oxygen saturation, more severe inflammation, higher D-dimer concentrations, and were more commonly prescribed antibiotics. The AUC for in-hospital mortality was for adADL: 0.7417 (95% CI: 0.6478–0.8357; *p* < 0.0001) and for ADL-change: 0.6869 (95% CI: 0.579–0.7928; *p* = 0.0018). The corresponding cut-offs were 0 for adADL and 3 for ADL-change. Cox proportional hazard models yielded hazard ratios of 3.57 (95% CI 1.57–8.10; *p* = 0.0024) for adADL and 3.78 (95% CI 1.49–9.54; *p* = 0.005) for ADL-change. ADL assessment offers valuable insights into in-hospital mortality among older COVID-19 patients. Monitoring ADL in these patients indicates high-risk individuals for in-hospital death. Integrating ADL into routine clinical practice might enhance care for older patients.

## 1. Introduction

Independence allows older adults to maintain their dignity, reduce the burden on caregivers, and avoid the negative psychological effects of dependency [1,2]. To maintain independence and manage daily living, individuals must perform various activities, such as dressing, eating, drinking, and personal hygiene. Unfortunately, aging often increases reliance on others for these and many other essential functions.

Activities of Daily Living (ADL) are fundamental tasks individuals must perform to manage their basic physical needs [3]. ADL can be assessed using standardized tools and is focused on bathing, dressing, using the toilet, eating, controlling physiological functions, and mobility (e.g., getting out of bed or walking). Each activity is assessed individually based on the ability to perform them independently. ADL measurement is simple and fast. Higher scores on ADL assessments indicate greater independence and better functional capacity. DL assessments are straightforward and quick to administer. Assessing ADL helps healthcare providers determine the level of assistance chronically ill and/or older adults require, guiding care planning and support [4].

The clinical value of ADL is well-documented [5]. ADL dependence is associated with not only a greater likelihood of institutionalization [6] and a higher risk of mortality [7,8] but also increased healthcare costs [9].

SARS-CoV-2 infection triggered a worldwide pandemic of COVID-19 disease. It significantly impacted older adults, leading to greater social isolation, increased health complications, and a need for targeted healthcare and support strategies. It also caused higher general and in-hospital mortality among older people [10]. Anemia and severe inflammation are, among others, important factors that increase in-hospital mortality [11,12].

Several factors have been identified as predictors of COVID-19 mortality, including age, comorbidities, inflammatory markers, anemia, or thrombosis. However, the prognostic value of pre-existing functional status, as assessed by ADL, remains largely unexplored. COVID-19 can have significant and long-lasting impacts on multiple organ systems, leading to functional decline and increased dependence.

The COVID-19 pandemic contributed to a decline in older adults’ ability to perform basic tasks independently due to the physical and cognitive effects of the virus. Social isolation worsens mental health issues, such as anxiety, loneliness, and reduced physical activity, leading to weight gain and an increased risk of conditions like diabetes and hypertension [13,14]. Older adults, often unfamiliar with technology, were forced to rely on teleconsultations instead of in-person visits, further limiting access to care [15]. Misinformation and a lack of understanding of the disease amplified their isolation, causing decreased motivation, cognitive decline, and worsening health [16]. Even after recovery, many continued to experience long-term symptoms or recurrent infections.

A decline in patient function is typically associated with increased comorbidities, complications, and mortality risks. ADL holds prognostic potential for various clinical outcomes. However, the predictive value of ADL for in-hospital mortality remains uncertain. While some studies suggest an association between poor ADL performance and increased mortality [17,18], others do not [19,20].

We hypothesize that the assessment of ADL at the time of hospital admission and before hospitalization can identify older patients who are at an increased risk of in-hospital mortality due to COVID-19. Specifically, we assumed that poor performance on ADL might be a significant predictor of adverse outcomes in this population.

Our retrospective analysis explored the usefulness of examining ADL at admission and the pre-COVID-19 to admission ADL difference (ADL change) as potential predictors of in-hospital mortality.

## 2. Materials and Methods

### 2.1. Study Design and Patients

This observational, cross-sectional study retrospectively utilized data from 141 consecutive elderly patients aged at least 65 years with COVID-19 who were admitted to the Neurology Ward at MSWiA Hospital in Poznań, Poland. This ward was transformed into a COVID-19-dedicated unit from 10 September 2021 until 15 January 2022, when the delta variant of the COVID-19 virus dominated. Details of retrospective analysis are presented in the data collection section.

Due to the study’s retrospective nature, the Bioethical Committee at the Poznan University of Medical Sciences, Poznan, Poland, reviewed and approved the study protocol and granted its permission to proceed (550/24). This Committee also waived the need for informed consent. The research was conducted following the Declaration of Helsinki [21]. Each patient was assigned a unique code to ensure their anonymity and the confidentiality of sensitive and clinical data.

All patients were tested positive for COVID-19. Admitting decisions were made upon a triage performed by experienced internal medicine specialists based on clinical presentation and laboratory/imaging results. The following patients with mild, moderate, and severe COVID-19 (Grades I–III on the 4-point scale according to the NIH COVID-19 Treatment Guidelines) [22] were eligible for admission to our Ward.

Grade I (mild): these were patients primarily presenting with fever, cough, sore throat, loss of smell or taste but without signs of pneumonia or respiratory distress.

Grade II (moderate): Patients in this category exhibited symptoms of pneumonia visible on imaging studies and maintained oxygen saturation (SpO_2_) ≥ 94% while breathing ambient air.

Grade III (severe): Characterized by oxygen saturation (SpO_2_) < 94%, respiratory rate ≥ 30 breaths/min, or a PaO_2_/FiO_2_ ratio < 300 mmHg. This group included patients with extensive lung involvement (e.g., >50% of lung parenchyma affected) and required hospitalization and intensive monitoring.

Patients with critical disease (Grade IV) requiring mechanical ventilation, ECMO, or treatment for acute respiratory failure, septic shock, or multi-organ failure were directly admitted to the intensive care unit and excluded from this study.

### 2.2. Data Collection

Data collected included demographic information, current health status, and laboratory test results. Previous neurodegenerative and cognitive disorders, stroke, ischemic heart disease, cardiac failure, hypertension, asthma/chronic obstructive pulmonary disease, diabetes, and kidney disease were determined based on information provided by patients or their relatives (including medication lists, clinical records, and detailed medical history).

Laboratory data included leukocyte count and hemoglobin, d-dimer, C-reactive protein, and procalcitonin concentrations. High-resolution computed tomography (HRCT) or chest radiographs assessed lung involvement. COVID-19 treatment data included types of antiviral medication, use of antibiotics, thromboembolic prophylaxis, and oxygen therapy.

### 2.3. Measuring ADL

The ADL scale is a simple tool that does not require special training or specific instruments. It is based on information obtained from the patients or their families. ADL was assessed by nursing staff using the Katz Index, which includes six basic functions: bathing, dressing, toileting, transferring, continence, and feeding [23]. Each function is scored as independent (1 point) or dependent (0 points), resulting in a total score ranging from 0 to 6. While an ADL score of 6 indicates complete functional independence, a score of 0 signifies total disability and reliance on others. Lower scores suggest needing assistance. The functional independence in ADL was evaluated at two time points:

- adADL: ADL measured upon hospital admission.
- Pre-COVID-19 ADL: ADL measured retrospectively based on clinical history before hospitalization.

ADL change was calculated using these values as the difference between pre-COVID-19 ADL and admission ADL.

### 2.4. Statistical Analysis

Since most continuous or discrete data did not follow a normal distribution (Q-Q plots and the D’Agostino-Pearson test), results are presented as median and 25th and 75th percentiles (Q1 and Q3, respectively). The Mann–Whitney test compared these data between survivors and non-survivors. The Fisher’s exact test was used to compare the proportions of binomial data between survivors and non-survivors. Receiver Operating Characteristic (ROC) curves examined the relationship between ADL and in-hospital mortality, with Youden’s index establishing the cut-off point for identifying high-risk patients. Univariate Cox proportional hazard models adjusted for age and gender were used to study the predictive value of ADL parameters for in-hospital death. As the number of non-survivors was 30, we could not use more than three variables in the Cox proportional hazard models. Hazard ratios and ROCs are presented with 95% confidence intervals (95% CI). A *p*-value < 0.05 was considered significant. PQStat v.1.8.6.120 (PQStat Software, Poznań, Poland) was used for statistical analyses.

## 3. Results

Thirty patients (21.3% of all) died during hospitalization. The median time to death was six days (4–11), significantly shorter than the hospital stay duration for survivors (12; 10–14 days). Table 1 summarizes comparisons of continuous/discrete data and qualitative data between survivors and non-survivors.

Non-survivors were older than survivors by nine years and had slightly lower pre-COVID-19 ADL. Their median admission ADL score was 0, and the ADL change was 2. Additionally, non-survivors exhibited lower oxygen saturation (sO2) by 3%, more severe inflammation, higher D-dimer concentrations, and were more commonly prescribed antibiotics (by over 32%).

The AUC for in-hospital mortality was as follows:

- Pre-COVID-19 ADL: 0.6098 (95% CI: 0.6478–0.8357; *p* = 0.0656—not significant, thus no cut-off was determined and further studied);
- Admission ADL: 0.7417 (95% CI: 0.6478–0.8357; *p* < 0.0001);
- ADL change: 0.6869 (95% CI: 0.579–0.7928; *p* = 0.0018).

The corresponding cut-offs were 0 for admission ADL and 3 for ADL change, i.e., the reduction in ADL by at least 3 points. Cox proportional hazard models yielded hazard ratios:

- 3.57 (95% CI 1.57–8.10; *p* = 0.0024) for admission ADL;
- 3.78 (95% CI 1.49–9.54; *p* = 0.005) for ADL reduction by at least 3 points.

Before admission, 3 patients (2.1%) had an ADL score of zero, while 84 (59.6%) had a perfect score of 6. At admission, the number of patients with an ADL score of zero increased to 49 (34.8%), whereas 25 (17.7%) maintained a score of 6. Additionally, a decline of at least 3 ADL points from pre-hospitalization levels was observed in 65 (46.1%) patients.

Compared to surviving patients, non-survivors were older by nine years and had slightly lower pre-COVID-19 ADL. Their median admission ADL score was 0, and the ADL change was 2. Additionally, non-survivors exhibited lower oxygen saturation (sO2) by 3%, more severe inflammation, higher D-dimer concentrations, and were more commonly prescribed antibiotics (by over 32%).

## 4. Discussion

We report that poor ADL at admission and an ADL decline of more than 2 points (compared with pre-COVID-19 infection) are associated with an increased risk of in-hospital death in older COVID-19 patients, regardless of age and sex.

Self-care tasks significantly impact older patients’ quality of life, directly affecting their ability to live independently and meet basic needs [3,4,5,17]. Difficulties with ADL correlate with adverse health outcomes, including increased morbidity and premature mortality across different groups and diseases. However, data specific to COVID-19 patients and in-hospital mortality remain limited.

Bruno et al. [17] conducted a multicenter, international study in 2359 elderly COVID-19 patients who were 70 years or older admitted to intensive care units (ICUs). Most patients (80%) were independent before hospitalization with Katz ADL of 6. Reduction of ADL < 6 was a significant predictor of increased risk of death. ICU death was observed in 62% of all patients. Their high-risk group with ADL < 6 separated those who were completely independent from the remaining older people.

Our single-center study involved only 141 older patients, presenting substantial differences compared to Bruno et al. [17]. Firstly, a smaller proportion of our patients were completely independent before hospitalization (59.6% vs. 80%). Secondly, our patients were younger at admission, with a minimum age of 65 compared to 75 years in Bruno et al.’s study. Thirdly, our ward was not an ICU. Additionally, our ADL cut-offs differed; we focused on complete disability (ADL score of 0) and a decline of at least 3 ADL points, while Bruno et al. considered any disability (ADL score < 6). Finally, our mortality rate of 21.3% was considerably lower than the 62% ICU mortality reported by Bruno et al. All in all, it appears that Bruno et al.’s patients were likely more severely ill, as demonstrated by the higher mortality rate and the fact that all participants were ICU patients. In contrast, our cohort represented a less critically ill population that did not require ICU-level care upon admission.

Other studies also assessed ADL in COVID-19, mainly at admission to the hospital [18,19]. However, they did not examine the interaction between COVID-19 and ADL.

ADL can be measured retrospectively for the pre-hospital phase and nearly instantly at admission to the hospital, and ADL changes between these two time points. Whereas pre-hospital ADL did not show any predictive value in our study, both the admission ADL of 0 and ADL change of more than 2 identified older patients at higher risk for in-hospital mortality.

Our analysis identified significant cut-off points for ADL scores in predicting mortality among hospitalized elderly patients with COVID-19. Nearly 24% of our patients were completely disabled upon admission, with an ADL score of zero, and faced a 3.5-fold increased risk of death. Approximately one-third of the cohort experienced a decline of at least 3 ADL points from their pre-COVID-19 levels, resulting in a 3.7-fold higher mortality risk. These findings underscore the clinical relevance of ADL assessment.

COVID-19 has had a profound impact on the health and functional status of all people, particularly those with multiple comorbidities and vulnerable populations, such as older adults with poor ADL.

## 5. Study Limitations

Our study has several limitations. It is a retrospective, single-center study, which limits the generalizability of our findings. We did not have control groups of patients of comparable age who were not admitted to the hospital at all or those who required ICU admission. This lack of control groups may affect the robustness of our conclusions. Another limitation is the absence of validated ADL cut-offs for in-hospital mortality. With only 30 deaths, our Cox proportional hazard model adjustment could reliably include only two covariates. However, even after adjusting for additional covariates such as hypertension, diabetes, previous TIA or stroke, cardiovascular disease, renal failure, neurodegenerative diseases, and chronic pulmonary disease or asthma, both admission ADL and ADL change remained significant predictors of in-hospital mortality.

We also studied multicollinearity between ADL at admission or ADL change and continuous variables. Only age and biochemical parameters were significantly but weakly correlated (rho between −0.19 and −0.39), which does not indicate multicollinearity. Nevertheless, we built several Cox proportional models with these parameters, and in all of them, ADL at admission or ADL change remained significant predictors of in-hospital mortality. However, with so many variables, these analyses might be considered methodologically flawed and were performed only to understand the potential impact of other factors. Therefore, the results of these exploratory analyses are not shown here.

Our study did not clearly define the time frame for assessing pre-existing ADL, and we assumed it reflected just a general patient’s functional status before acute COVID-19. Additionally, all our patients were Poles, entailing European ethnicity, limiting our findings’ applicability to other ethnic groups.

While COVID-19 incidence has decreased, it remains a significant cause of in-hospital mortality, underscoring the need for risk stratification among elderly patients. Another global infectious disease pandemic could emerge at any time. The experience gained from COVID-19 has provided valuable insights into caring for older and vulnerable individuals. In emergencies, simple solutions and tools often prove most effective. ADL is such a tool, offering substantial clinical value beyond assessing older people’s functional status.

A key strength of our study is the simplicity of ADL assessment using the Katz Index, which any member of the therapeutic team can perform. As we move beyond the pandemic, measuring ADL remains crucial for identifying older individuals at risk of premature death. Monitoring ADL, especially when significantly reduced, can guide targeted interventions and ensure close monitoring.

## 6. Clinical Implications

ADL assessment seems to be a valuable tool for predicting in-hospital mortality in elderly COVID-19 patients. This straightforward method helps identify high-risk patients, enabling timely and targeted interventions. Healthcare providers can better allocate resources and develop tailored care plans to improve patient outcomes by focusing on ADL changes. Monitoring ADL in hospitalized patients also aids in adjusting care strategies to enhance recovery and reduce mortality rates. Further prospective studies are necessary to establish the definitive role of ADL in clinical practice.

## 7. Conclusions

This study demonstrates that poor ADL at admission and a decline of more than 2 ADL points are significant predictors of in-hospital mortality in older COVID-19 patients. ADL assessment offers valuable insights into in-hospital mortality among these patients, emphasizing its clinical utility beyond functional status evaluation. Monitoring ADL upon hospital admission provides a straightforward method to identify high-risk individuals, allowing for timely and targeted interventions to improve patient outcomes.

COVID-19 has been associated with severe respiratory issues, cardiovascular complications, and long-term symptoms such as fatigue and cognitive impairment, which further impair the ability to perform ADL. Complete functional disability or a significant decline in ADL identifies those at higher risk for in-hospital death. Integrating ADL assessment into routine clinical practice can enhance elderly patient care by guiding targeted interventions and ensuring close monitoring.

Despite the limitations of our single-center, retrospective study, our findings highlight the importance of ADL assessment in the early identification of high-risk patients and the need for personalized care strategies. Further prospective studies are necessary to establish the definitive role of ADL in clinical practice.

## Figures and Tables

**Table 1:** Comparison of clinical characteristics between surviving and non-surviving hospitalized elderly (Age ≥ 65 years) patients with COVID-19.

	Survivors N = 111			Non-Survivors N = 30			
Continuous or Discrete Data							
	Median	Q1	Q3	Median	Q1	Q3	*p*-Value ^#^
age [years]	75	69	83	84	74	89	0.0043
pre-COVID-19 ADL	6	5	6	5	4	6	0.0377
Admission ADL	3	0	5	0	0	2	<0.0001
ADL change	2	0	4	4	3	5.25	0.0016
CLINICAL DATA							
Pulse rate [beats/min]	80	72	96	86.5	74.5	97	0.4081
Skin temperature [Co]	36.5	36.4	36.8	36.5	36.2	36.7	0.1492
Systolic blood pressure [mmHg]	140	119	152	135.5	112.75	150	0.7128
Diastolic blood pressure [mmHg]	78	66	87	76.5	70	84.5	0.8127
sO2 [%]	93	90	95	90	84.5	93.5	0.0119
LABORATORY DATA							
Hemoglobin concentration [g/dL]	12.8	12.1	14.3	12.15	10.95	13.4	0.1268
WBC count [10^9^/l]	8.7	6.8	11.6	11.2	8.5	14.3	0.0097
C-reactive protein concentration [mg/L]	61.5	31.5	116.7	147.0	89.0	207.2	0.0002
Procalcitonin concentration [ng/mL]	0.14	0.08	0.27	0.52	0.27	1.77	<0.0001
D-dimer concentration [µg/mL]	1.2	0.7	3.7	3.7	1.1	11.5	0.0049
Qualitative Data							
	N	%		N	%		*p*- value *
Male sex	48	43.2		14	46.7		0.8364
DATA FROM MEDICAL HISTORY							
Anti-SARS-CoV-2 vaccination	60	54.1		10	33.3		0.0631
Ischemic heart disease	20	18.0		5	16.7		1
Heart failure	8	7.2		6	20.0		0.077
Hypertension	71	64.0		18	60.0		0.6769
Renal failure	13	11.7		4	13.3		0.7591
Neurodegenerative disease	19	17.1		8	26.7		0.295
past stroke-TIA	23	20.7		8	26.7		0.4674
Diabetes	40	36.0		11	36.7		1
Asthma or COPD	15	13.5		2	6.7		0.5268
IN-HOSPITAL COVID-19-RELATED THERAPY							
Antiviral treatment against SARS-CoV-2	61	55.0		11	36.7		0.0996
Antibiotic treatment	64	57.7		27	90.0		0.001

^#^—comparisons made by the Mann–Whitney test. *—comparisons made by the Fisher exact test. Abbreviations: pre-COVID-19 ADL change—the difference between the pre-COVID-19 and the admission ADL—Activities of Daily Living score; COPD—chronic obstructive pulmonary disease; Q1—25th percentile; Q3—75th percentile; TIA—transient ischemic attack.

## Data Availability

The datasets used and/or analyzed during the current study are available from the corresponding author upon reasonable request.
